# Non‐Invasive 3D Photoacoustic Tomography of Angiographic Anatomy and Hemodynamics of Fatty Livers in Rats

**DOI:** 10.1002/advs.202205759

**Published:** 2022-11-17

**Authors:** Xin Tong, Li Lin, Peng Hu, Rui Cao, Yang Zhang, Joshua Olick‐Gibson, Lihong V. Wang

**Affiliations:** ^1^ Caltech Optical Imaging Laboratory Andrew and Peggy Cherng Department of Medical Engineering Department of Electrical Engineering California Institute of Technology Pasadena CA 91125 USA; ^2^ Present address: College of Biomedical Engineering and Instrument Science Zhejiang University Hangzhou 310027 China

**Keywords:** hepatic steatosis, liver imaging, non‐alcoholic fatty liver, photoacoustic computed tomography

## Abstract

Non‐alcoholic fatty liver disease is the most common liver disorder worldwide, which strongly correlates to obesity, diabetes, and metabolic syndromes. Complementary to mainstream liver diagnostic modalities, photoacoustic tomography (PAT) can provide high‐speed images with functional optical contrast. However, PAT has not been demonstrated to study fatty liver anatomy with clear volumetric vasculatures. The livers of multiple rats are non‐invasively imaged in vivo using the recently developed 3D PAT platform. The system provides isotropically high spatial resolution in 3D space, presenting clear angiographic structures of rat livers without injecting contrast agents. Furthermore, to quantitatively analyze the difference between the livers of lean and obese rats, the authors measured several PAT features and statistical differences between the two groups are observed. In addition to the anatomy, a time‐gated strategy is applied to correct respiration‐induced motion artifacts and extracted the hemodynamics of major blood vessels during the breathing cycles. This study demonstrates the capabilities of 3D‐PAT to reveal both angiographic anatomy and function in rat livers, providing hematogenous information for fatty liver diagnosis. 3D‐PAT, as a new tool for preclinical research, warrants further improvements to be transferred to human pediatric liver imaging.

## Graphical abstract

In this work, a time‐gated strategy is applied in a 3D photoacoustic tomography (PAT) platform to non‐invasively image the livers of multiple rats in vivo. PAT features are developed to compare the difference between the livers of lean and obese rats. Other than motion‐free anatomy, the time‐gated strategy helps extract the hemodynamics of major blood vessels during respiration cycles.

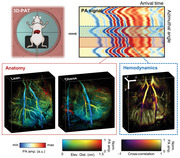

## 1 Introduction

Non‐alcoholic fatty liver disease (NAFLD), also termed metabolic (dysfunction) associated fatty liver disease, is the most common chronic liver disease in developed countries. In the United States, approximately 30% of the population has been affected, with projected total affected cases of 100 million in 2030.^[^ ^1^, ^2^, ^3^, ^4^ ^]^ NAFLD includes a spectrum of histopathological symptoms ranging from hepatic steatosis to non‐alcoholic steatohepatitis (NASH), which may further develop into fibrosis, cirrhosis, or hepatocellular carcinoma.^[^ ^5^ ^]^ People with NAFLD have a higher risk for certain hematogenous issues, including cardiovascular disease, high blood pressure, and abnormal levels of fats in the blood.^[^ ^1^ ^]^ There remain heterogeneous theories of the pathogenesis of NAFLD, and accurate and early diagnosis is critical to treating the disease.^[^ ^6^, ^7^ ^]^

Diagnostic techniques for assessing NAFLD include biopsy, magnetic resonance imaging (MRI), ultrasound (US), and X‐ray computed tomography (CT).^[^ ^1^, ^3^, ^8^, ^9^, ^10^ ^]^ Conventional US is widely adopted clinically for NAFLD diagnosis due to its low cost and mobility.^[^ ^11^ ^]^ Recent advances in US include shear wave elastography to measure liver stiffness,^[^ ^12^ ^]^ as well as ultrafast power Doppler imaging^[^ ^13^, ^14^ ^]^ and ultrasound localization microscopy^[^ ^15^ ^]^ to reveal angiographic details, while they either rely on invasive microbubble injection or require longer scanning time for volumetric angiograms with a large field of view (FOV). X‐ray CT provides images with high spatial resolution and deep penetration, while the modality is limited by radiation exposure and relatively poor contrast of soft tissue. MRI, including magnetic resonance elastography, is widely applied to NAFLD diagnosis due to its high accuracy by spectroscopy or proton density fat fraction, while MRI requires a longer scanning time, higher costs, and is unsuitable for patients with certain implants.^[^ ^16^, ^17^, ^18^, ^19^ ^]^ Many other non‐invasive modalities emerged to access liver diseases, such as diffusion‐weighted imaging and deep learning‐assisted methods, while none of them can distinguish NAFLD from the other liver diseases individually with as high confidence as biopsy.^[^ ^20^, ^21^, ^22^ ^]^ Liver biopsy is now the most accurate method for detecting steatohepatitis and fibrosis in NAFLD patients, but it is well recognized to be restricted by expense, sample error, and procedure‐related mortality and morbidity.^[^ ^1^ ^]^ Therefore, there has been great interest in establishing more non‐invasive biomarkers for the diagnosis of the NAFLD spectrum.

Combining acoustic detection with optical function contrasts, photoacoustic tomography (PAT) is a rising imaging modality to provide high‐resolution, speckle‐free images with deep penetration in biological tissue.^[^ ^23^, ^24^ ^]^ The rich optical absorption contrast from various endogenous and exogenous agents enables PAT to perform structural, functional, and molecular imaging.^[^ ^25^ ^]^ PAT relies on the generation and detection of photoacoustic (PA) waves. The PA waves are emitted due to transient thermoelastic expansion caused by the irradiation of a short‐pulsed laser on biological tissues within the safety limit.^[^ ^26^ ^]^ For detection, ultrasonic transducers are arranged around the region of interest (ROI) with various configurations, and the optical absorbers’ distribution is reconstructed from the measured PA signals.^[^ ^27^ ^]^ The versatility of PAT to image different animal and human subjects has been demonstrated through extensive preclinical and clinical trials.^[^ ^27^, ^28^, ^29^ ^]^

Small animal whole‐body PAT has been shown to reveal the liver structure.^[^ ^28^, ^30^, ^31^ ^]^ PAT also has been combined with other modalities to diagnose steatosis and fibrosis.^[^ ^32^, ^33^, ^34^, ^35^ ^]^ These applications have demonstrated PAT as highly complementary to the mainstream hepatic imaging modalities. However, difficulties exist in previous studies to reveal detailed volumetric angiographic structures (i.e., individual blood vessels) of the liver in vivo with high speed and without exogenous contrast agents due to system limitations such as insufficient spatial sampling rate and limited detection angle. We have recently developed a high‐speed three‐dimensional PAT (3D‐PAT) system capable of capturing volumetric PA images with isotopically high spatial resolution.^[^ ^27^ ^]^ After applying respiration‐gated motion correction, the breathing‐induced artifacts have been effectively removed, and clear liver angiographic details have been presented. Moreover, we imaged multiple lean and obese rats and performed quantitative comparisons on their livers based on selected structural properties, namely vessel volume occupancy, vessel number density, angiographic irregularity, and estimated speed of sound. Statistical differences between the two groups indicated the structural difference in fatty livers from lean ones. Other than anatomical details, the time‐gated frames render the dynamic volumetric image sequence, which clearly reveals the anatomical deformation and hemodynamics of large blood vessels. This study presents great potential of PAT in assessing fatty livers, thus contributing to the diagnosis of NAFLD with additional information.

## 2 Results

### 2.1 3D‐PAT of the Rat Liver Anatomy

**Figure 1:**
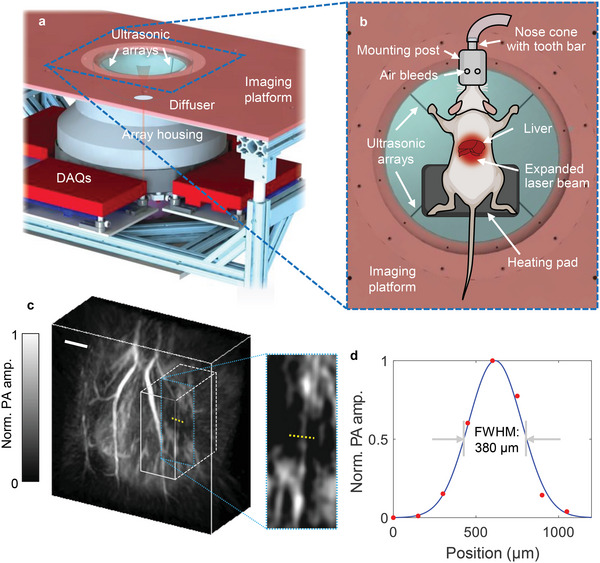
Schematics and performance of the 3D‐PAT system. a) Schematic of the 3D‐PAT imaging system. The 1064 nm laser beam is expanded by a diffuser fixed on the bottom of the hemisphere. b) Close‐up view of the imaging aperture for rat experiments. The rat is placed on a heating pad with its liver at the center of the expanded laser beam. Two of the four arc‐shaped ultrasonic transducer arrays are marked. c) Orthogonal projection of a rat liver imaged by 3D‐PAT. The close‐up shows a slice within the volume with a blood vessel selected for resolution evaluation. Scale bar, 5 mm. d) PA amplitude profile along the yellow dotted line in (c), representing a full width half maximum (FWHM) of 380 µm.

The schematic of the 3D‐PAT system for liver imaging is shown in **Figure** **1**a. A more detailed description of the system can be found in Section 4 and Figure S1, Supporting Information. Four arc‐shaped ultrasonic transducer arrays (256 elements in each) are integrated into a hemispherical housing, which can be rotated coaxially to provide a densely sampled detection matrix. The elements are connected to a pre‐amplification circuit and four data acquisition (DAQ) modules. For illumination, the diffused laser beam (Nd:YAG laser, 50 Hz repetition rate) is directed to the subject through the bottom of the array housing. During imaging procedures, the rat was placed on a heating pad on top of the arrays, with its liver near the hemisphere center, as shown in Figure 1b. A 3D‐printed nose cone with a tooth bar was used to guide the air for breathing. The hairs around the abdomen area were removed, with the hepatic region placed at the center of the expanded laser beam.

The one‐way scanning took 10 s to finish, while round‐trip scanning is preferred for time‐gated reconstruction with dense sampling. Motion artifacts induced by respiration and heartbeat were observed in the raw signal as oscillatory stripes (as shown in Figure S2, Supporting Information), blurring the reconstructed image. We applied time gating (TG) by removing the motion‐affected frames and combining the round‐trip signals before reconstruction to enhance the image quality (see Section 4 and Figure S2, Supporting Information, for more details). Images acquired with/without respiration/heartbeat TG and with/without round‐trip scanning are compared in Figure S3, Supporting Information.

**Figure 2:**
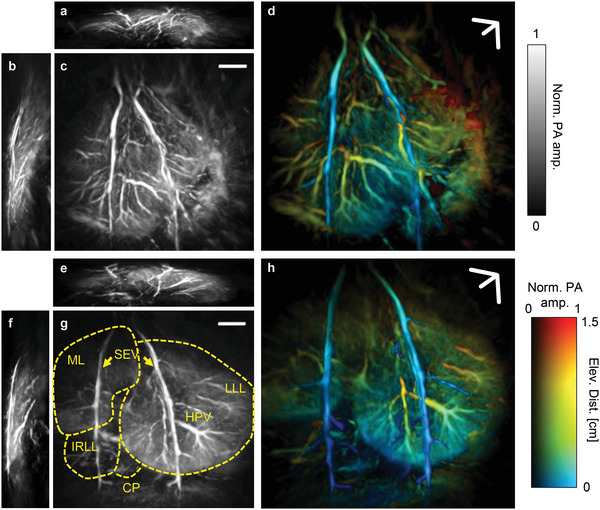
3D‐PAT of lean and obese rat livers in vivo. Maximum amplitude projections (MAPs) of a lean rat liver in a) axial, b) sagittal, and c) coronal views. d) Coronal perspective projection of the lean rat liver angiogram with depth encoding. MAP images of an obese rat liver in e) axial, f) sagittal, and g) coronal views. ML, median lobe. LLL, left liver lobe. IRLL, inferior right lateral lobe. CP, caudate process. SEV, superior epigastric vessels. HPV, hepatic portal veins. h) Coronal perspective projection of the obese rat liver angiogram with depth encoding. Scale bars, 5 mm.

Using 3D‐PAT with TG motion correction, we obtain high‐quality, motion‐artifact‐free volumetric images in vivo, visualizing the vasculature down to an apparent vascular diameter of 380 µm shown in Figure 1c,d. We present the maximum amplitude projections (MAPs) of a lean and obese rat liver, together with the color‐encoded perspective projection renderings for depth information, as shown in **Figure** **2**. At 1.2 cm depth below the liver surface, the contrast‐to‐noise ratio (CNR) of a blood vessel with diameter > 400 µm is ≈5. Since oxyhemoglobin dominates the optical absorption at 1064 nm wavelength, PAT renders detailed hepatic angiograms. As denoted in Figure 2g, the median lobe (ML), left liver lobe (LLL), inferior right lateral lobe (IRLL), and many blood vessels, including the superior epigastric vessels (SEV), hepatic portal veins (HPV), and their branches are highlighted.

### 2.2 Quantitative Comparison between Lean and Obese Rat Livers

**Figure 3:**
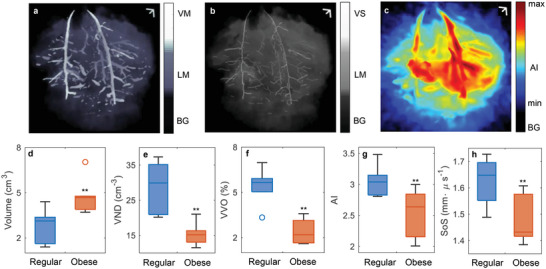
Quantitative comparison between lean and obese rat livers. a) Coronal perspective projection of the binary liver mask and vessel segmentation mask. BG, background; LM, liver mask; VM, vessel mask. b) Coronal perspective projection of the binary liver mask and vessel skeleton. VS, vessel skeleton. c) Perspective projection of AI with the binary liver mask. Statistical comparison of d) liver mask volume, e) VVO, f) VND, g) AI, and h) estimated SoS between lean and obese rat livers. Data are presented as box plots; *p*‐values are calculated using one‐tailed Welch's (unequal variances) *t*‐tests. **p < 0.01. Scale bars, 5 mm.

Based on the same configuration, we repeated the imaging procedure on multiple Zucker obese and lean rats (as control groups). The Zucker obese rats are genetically obese models with metabolic‐associated hepatic steatosis.^[^ ^6^, ^36^ ^]^ Using the motion‐corrected volumetric PAT images, we selected five features: liver mask volume, vessel volume occupancy (VVO), vessel number density (VND), angiographic irregularity (AI), and estimated speed of sound (SoS), to investigate the structural difference between healthy and obese rat livers, as shown in **Figure** **3**.

Some parameters require the binary masks of the liver and major blood vessels (CNR greater than 3). We applied adaptive thresholding on the raw and vessel‐enhanced volumetric images^[^ ^37^ ^]^ to segment the liver and large blood vessels separately, as shown in Figure 3a (see Section 4 for more details). The process was repeated on all rats (*n* = 6 in each group), and we compared the liver mask volume of normal and obese rats to acquire the boxplots, as shown in Figure 3d. To determine if the differences between the lean and obese rat livers were statistically significant, we applied the one‐tailed Welch's (unequal variances) *t*‐test to determine the *p*‐value against the null hypothesis *H*_0_ that the mean mask volume of the obese rat livers is no larger than that of lean rats. The resulting *p*‐value was smaller than 0.01, indicating a rejection of *H*_0_ at the 1% significance level.

We defined VVO as the volume ratio between the binary vessel segmentation mask and the binary liver mask, which represents the relative richness of the major blood vessels. We also calculated VND, defined as the number density of the vessel skeletons within the liver mask. To extract the vessel skeleton (as shown in Figure 3b), we extracted blood vessel skeletons by generating vessel centerlines from the vessel segmentation masks as mentioned above and divided the number of individual blood vessels by the mask volume to acquire the total VND (see Section 4). Similar statistical analysis was then performed on VVO and VND, and significant differences (*p* < 0.01) were also observed, as shown in Figures 3e,f. Both VVO and VND showed significant statistical differences between lean and abnormal rat livers, indicating a decrease in vessel richness in the fatty livers, possibly due to the fat accumulation and steatosis on the liver surface, especially near the hepatic and portal vein regions.^[^ ^5^ ^]^ As an extreme case, a notable reduction of vessel density in the liver caused by angiogenesis has been observed in experimental hepatocellular cancer models.^[^ ^38^ ^]^

**Figure 4:**
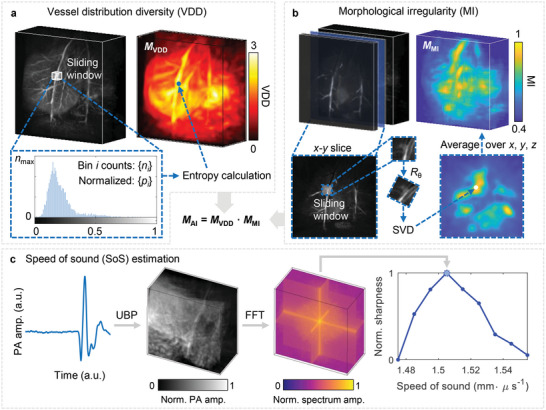
Schematics of calculating the angiographic irregularity (AI) and speed of sound (SoS) estimation. a) Schematics of vessel distribution diversity (VDD) calculation. A sliding window is scanned across the 3D image. At each position, the image histogram is acquired, and the entropy is calculated based on the normalized histogram counts. The value is then assigned to the center of the scanning window to form a 3D map *M*_VDD_. b) Schematics of morphological irregularity (MI) calculation. At each *x*–*y* slice of the 3D image, a sliding window is scanned across the slice. The window is rotated from 0° to 180°, and the dominant normalized singular value is recorded at each angle. The MI at the window center is assigned according to the biggest difference in the dominant normalized singular value of all angles. Similar processes are repeated over all *y*‐*z* and *x*‐*z* slices to form the 3D map *M*_MI_. The AI is calculated as the dot product of the two maps. c) Schematics of the speed of sound (SoS) estimation. The raw PA signal is universally back‐projected (UBP) with different SoSes in tissue to form the image, and 3‐D fast Fourier transform (3D‐FFT) is performed to acquire the spectrum. The image sharpness is calculated as the mean of the spectrum amplitude. The value that maximizes the sharpness is chosen as the estimated SoS.

AI consists of two parameters: vessel distribution diversity (VDD) and morphological irregularity (MI), representing the value distribution and directional orientation of the image separately. The schematics of calculating the VDD and MI maps are shown in **Figure** **4**a,b, and more details can be found in Section 4. As in Figure 4a, a sliding window was scanned across the volumetric image for VDD calculation. We acquired the normalized image histogram within each window and calculated the VDD according to the information entropy. The VDD value was then assigned to the center of the scanning window to form a three‐dimensional VDD map. For MI calculation, as shown in Figure 4b, at each *x*–*y* slice of the 3D image, a sliding window was scanned across the slice, rotated from 0° to 180°, and decomposed through singular value decomposition (SVD). The MI was calculated based on the dominancy term among all the angles, and a similar process was repeated over all *y*–*z* and *x*–*z* slices to form the 3D MI map. We finally calculated the dot product of the two maps to get the angiographic irregularity map shown in Figure 3c. Statistical analysis was repeated and shown in Figure 3g with significant differences (*p* < 0.01).

Furthermore, it has been shown that livers suffering from NAFLD might have lower sound speeds than healthy livers.^[^ ^39^, ^40^, ^41^ ^]^ Speed of sound estimation using ultrasound has been extensively explored,^[^ ^42^, ^43^ ^]^ while precise measurement with PAT in vivo has been challenging due to the receiving‐only imaging mode. Here we propose an SoS estimation method in PA using the dual‐SoS universal back‐projection reconstruction algorithm.^[^ ^28^ ^]^ Within the region to be reconstructed, the water and tissue were separated by an ellipsoidal surface. By measuring the water temperature, we fixed the SoS in the water. Targeting a bulk of liver tissue at depth, we reconstructed the PAT image with different SoSes and evaluated the corresponding image sharpness. We then estimated the SoS averaged over the propagation distance and near the liver tissue, as shown in Figure 4c. Comparison between lean and obese rat livers gave a statistical difference with *p* < 0.01, as shown in Figure 3h. More details can be found in Section 4.

### 2.3 Respiration‐Gated Hemodynamics of Rat Liver

**Figure 5:**
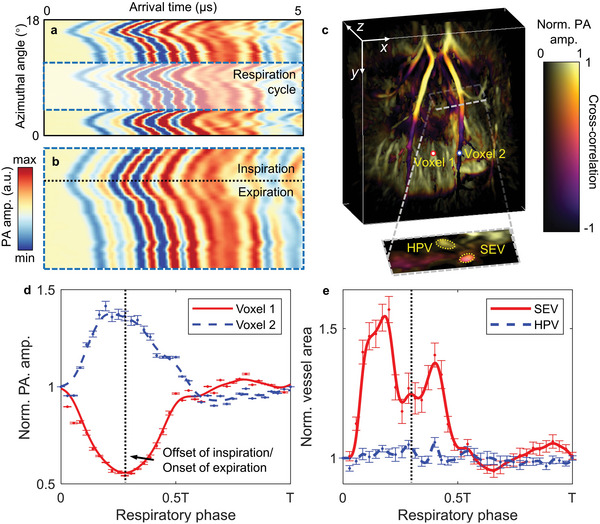
Respiration‐based time gating schematics and results. a) Schematic of the respiration‐based time gating for dynamic images. Image shows a part of raw PA signal from one transducer, where periodic oscillations appear as the result of breathing. b) Close‐up raw PA signal for one respiration cycle. Each cycle is divided into multiple phases, and each phase is picked up through all cycles before summing over round‐trip signals for reconstruction. c) Motion‐contrast‐encoded orthogonal projection of a rat liver with a slice showing cross‐sections of SEV and HPV. d) Relative changes of the PA signals from two voxels in c). Data are normalized according to the first phase and plotted as means ± standard errors of the mean. e) Relative changes of the cross‐sectional vessel areas in ©. Data are normalized according to the first phase and are plotted as means ± standard errors of the mean.

Other than anatomical structures, we can also utilize the motion‐affected frames to acquire dynamic images displaying periodic respiratory motion. To demonstrate the feasibility of 3D‐PAT to resolve breathing movements, we imaged a lean rat liver in light anesthesia. We divided the respiration cycle into multiple phases (as shown in **Figure** **5**a,b) and applied time‐gated reconstruction to acquire the dynamics frames of the liver angiogram, where periodic deformation in response to inspiration and expiration was observed (see Movie S1, Supporting Information).

From the raw signal, we observed a repetitive pattern composed of inspiration and expiration periods. By correlating this respiration pattern with the time sequence at each point on the volumetric image, we acquired a 3D motion‐contrast map, as shown in Figure 5c. We selected two voxels on the map and plotted their signal change over different respiration phases, as shown in Figure 5d. The two voxels have opposite patterns, highlighting their spatial separation along the *z*‐axis. More results can be found in Figure S4, Supporting Information. Moreover, on the axial slice shown in Figure 5c, we identified the cross‐sections of SEV and HPV. We then segmented the vessel cross‐sections using a two‐step thresholding method and calculated the cross‐sectional area changes over different respiration phases, as shown in Figure 5e (More details can be found in Section 4).

Figure 5c shows that PA amplitudes at the upper and lower parts of SEV have opposite variations, most likely because SEV goes through the diaphragm, which serves as a pivot for motion along the *z*‐axis, as shown in Figure S4, Supporting Information.^[^ ^44^ ^]^ The amplitude changes on the liver, on the other hand, are more uniform since the liver moves with the diaphragm as a whole. Figure 5e shows that SEV has more significant area changes than HPV. We observe an increase in SEV diameter during inspiration. This phenomenon, known as the respiratory pump, is characterized by negative intrathoracic pressure, which increases the venous return and the outgoing flow from the heart.^[^ ^45^ ^]^ The pump effect becomes less significant toward the lower SEV, as shown in Figure S5, Supporting Information. In contrast, area change in HPV remains smaller since HPV is further from the thoracic cavity, and the blood volume is less sensitive due to the extensive capillaries on the liver.^[^ ^46^ ^]^

## 3 Discussion and Conclusion

This study reports preclinical imaging results of the fatty liver using the recently‐developed 3D‐PAT system. We presented the anatomical volumetric renderings with high temporal resolution and imaging speed. Furthermore, we compared the structural differences in obese and lean rats’ livers, together with the statistical analysis of the vascular structure, angiographic irregularity, and estimated speed of sound. Lastly, we demonstrated the ability of the 3D‐PAT system to capture periodic motions using TG reconstruction and present the dynamic frames and hemodynamics in some major blood vessels of the liver during the respiratory cycle. This motion correction method is robust and flexible in other physiologically‐relevant distortions such as heartbeat.

We select five PAT features to compare lean and obese rat livers quantitatively and observe statistical significance in all of them. The features give a more comprehensive description of the liver, and different parameters may be suitable under different scenarios. VND and VVO are acquired based on the binary blood vessel masks and focus on the distribution of major vessels to capture global changes in the blood supply induced by fibrosis and cirrhosis.^[^ ^35^ ^]^ Compared to VVO, VND utilizes skeletonized vessel maps, which neglect the vessel thickness but keep the topology.^[^ ^47^ ^]^ VND thus warrants further investigation using fractal dimension and connectivity, which are closely related to cancerous tissue.^[^ ^48^, ^49^ ^]^ AI is acquired as a combined factor of entropy and anisotropy and is expected to be more accurate for early‐stage NAFLD diagnosis due to the following points. First, AI represents the distribution of the PA signal, which (at 1064 nm) mostly comes from hemoglobin in the major blood vessels and smaller ones, such as liver sinusoids. It has been shown that liver sinusoid‐related changes (such as capillarization) favor steatosis development and set the stage for NAFLD progression.^[^ ^50^ ^]^ Second, by tuning the illumination wavelength and using exogenous contrast agents, AI may capture NAFLD from various aspects. Third, instead of relying on whole‐liver masks, AI maps can be applied locally to help identify the damaged or diseased region in the liver (see Figure S4, Supporting Information).^[^ ^51^ ^]^ It is known that fatty livers have reduced speeds of sound, which serves as an important indicator for US diagnosis. By estimating the SoS in PAT, we expect to correlate with the US findings for cross‐validation. We also investigated other parameters, such as entropy (i.e., VDD in Figure 4a), anisotropy (i.e., MI in Figure 4b), and PA signal intensity, as shown in Figure S4, Supporting Information.

From the motion‐affected frames, 3D‐PAT can acquire dynamic volumetric movies simultaneously without additional cost. The feasibility study in hemodynamics is anticipated to benefit future studies in the following aspects. First, later stages of NAFLD, such as fibrosis and cirrhosis, might be associated with greater liver stiffness and portal hypertension.^[^ ^52^ ^]^ These factors can affect the morphological and functional dynamics of major blood vessels.^[^ ^53^ ^]^ 3D‐PAT's ability to detect respiration and heartbeat‐related hemodynamics might benefit the diagnosis in prospect. Moreover, NAFLD is known to be independently linked to cardiometabolic morbidity and mortality.^[^ ^54^ ^]^ Recent studies have also connected NAFLD with respiratory diseases such as chronic obstructive pulmonary disease (COPD),^[^ ^55^ ^]^ hypoxia,^[^ ^56^ ^]^ and asthma.^[^ ^57^ ^]^ 3D‐PAT of the liver thus warrants further investigation into cardiac and respiratory misfunctions using the time‐gated movies.

3D‐PAT complements established imaging modalities (such as US) by offering high‐speed volumetric imaging with scalable spatial resolution, various optical contrasts, and adaptable configurations. To further push the technique to benefit clinical diagnosis, the system warrants investigation in the following aspects. First, lasers with different wavelengths can be adapted to highlight various endogenous (e.g., lipid) and exogenous (e.g., indocyanine green) contrast agents in the liver. Second, PAT can easily hybrid with pulse‐receiver ultrasound measurement,^[^ ^58^, ^59^, ^60^ ^]^ thus improving the SoS estimation accuracy and introducing all the PAT features to enhance the specificity of standalone US diagnosis. Third, by imaging more livers with steatohepatitis, fibrosis, and cirrhosis, the 3D‐PAT system can employ structural parameter measurements and respiration‐gated techniques to address the deformation of different rodent livers, thus providing additional information for the diagnosis of the broader NAFLD spectrum. Lastly, more accurate reconstruction algorithms, such as the fast marching method,^[^ ^61^, ^62^ ^]^ can be adopted to further enhance the image quality.

Combining high spatiotemporal resolution and high sensitivity, 3D‐PAT provides hepatic anatomy and hemodynamics simultaneously without the need for ionizing radiation or exogenous contrast agents. With the following adjustments, it can be potentially translated to clinical studies, where the 2.25‐MHz center frequency is chosen to balance the FOV, the imaging depth, and spatial resolution for large rodent and human pediatric imaging. First, an ≈4 cm penetration depth has been demonstrated for human breast imaging, and the system is expected to achieve deeper penetration under the liver surface by utilizing different wavelengths (730 nm light is expected to achieve three times higher SNR at 5 cm depth than 1064 nm^[^ ^63^ ^]^), optimizing the illumination patterns (e.g., using donut beam illumination), and averaging over more scanning frames. Second, according to the spatial Nyquist sampling criterion,^[^ ^64^ ^]^ 3D‐PAT can cover an FOV of 16 cm with isotropic spatial resolution within 15 s, which is larger than the average liver size in adults.^[^ ^65^ ^]^ To enlarge the optical illumination area, we can use higher‐power lasers, join multiple lasers, or optimize the laser delivery paths.^[^ ^66^ ^]^ Third, the mechanical design of the imaging platform may be further modified to place the hepatic region closer to the optical window. In addition to the investigation of NAFLD, imaging and quantitatively measuring the angiographic environment in the liver using 3D‐PAT can serve as a test of response in drug development and treatment and as an alternative to invasive biopsies for diagnosis.

## 4 Experimental Section

### Experimental Setup

For ultrasonic detection, four arc‐shaped ultrasonic transducer arrays (Imasonic, Inc.; 2.25 MHz central frequency; >98% one‐way bandwidth; 4 × 256 elements) were connected to 32 sets of customized pre‐amplifiers and four DAQ modules (PhotoSound, Inc., ADC256; 40 MHz maximum sampling rate; 12‐bit dynamic range). The laser beam was expanded by an engineered diffuser (EDC‐15, RPC Photonics Inc.) sealed in the lens tube. The optical fluence on the tissue surface was limited by the American National Standards Institutes’ safety standards.^[^ ^67^ ^]^ 1064‐nm light was selected for illumination, which is the fundamental wavelength of the Nd:YAG laser (Model 9050DLS, 50 Hz rep rate, Powerlite, Continuum). To minimize the optical and acoustic energy loss, deuterium oxide (D_2_O, Isowater Corp.) was used to acoustically couple the holding cup (3–4 cm deep) with the ultrasonic arrays.

### Rat Liver Imaging Procedure

For lean liver imaging, adult, 9–10‐week‐old Zucker lean rats (Crl:ZUC‐*Lepr^fa^*, Charles River Laboratories, 150–200 g body weight) were used. For obese liver imaging, adult, 9–10‐week‐old Zucker obese rats (Crl:ZUC‐*Lepr^fa^*, Charles River Laboratories, 300–350 g body weight) were used. Before imaging, the rat was maintained under anesthesia with 1.5% vaporized isoflurane, and its eyes were covered by pharmaceutical‐grade ophthalmic ointment. The hair on the abdomen area was first removed using clippers and depilatory cream. Afterward, a 3D‐printed nose cone and tooth bar were placed on the rat to ensure its normal breathing. Then their limbs were fixed to metallic pillars mounted on the imaging platform using paper tapes to reduce the motion near the liver region.

### Image Reconstruction

To mitigate the artifacts induced by the acoustic inhomogeneity between the biological tissue and D_2_O, all the images were reconstructed using the GPU‐accelerated dual speed‐of‐sound universal back‐projection (DualSoS‐UBP) algorithm.^[^ ^27^, ^28^ ^]^ Specifically, since the rat abdomen region was placed at a piece of plastic wrap which also separated the D_2_O from the biological tissue, the entire region was segmented into two zones: a tissue zone and a D_2_O zone. It was assumed that the speed of sound was uniform within each zone but was different across the zones. To further simplify the problem, the following two assumptions were made. First, the cross‐section of the rat abdomen region was approximated by a half ellipse characterized by its center position and the lengths of its major and minor radii and fitted from the images reconstructed by single speed‐of‐sound UBP.^[^ ^68^ ^]^ Second, refraction at the boundary of the two zones was neglected, and ultrasonic rays traveled straight from the field point to the detectors. Based on these assumptions, the phase delay map between any source‐detector pairs could be calculated given the speed of sound in the tissue and D_2_O through line integration. Finally, the PA signals acquired at all scanning steps were back‐projected into the 3D space to form the volumetric image (voxel size 0.15  × 0.15 × 0.15 mm^3^). All the reconstructed images were further batch‐processed using a depth compensation method to enhance the PA amplitude in the deep tissue. A 3D high pass filtration and Hessian‐based Frangi vessel enhancement filtering^[^ ^37^ ^]^ were then applied to the images to enhance the contrast of blood. The filtered images (self‐normalized) were finally added back to the original depth‐compensated images and obtained the presented images. The PHOVIS software developed in MATLAB^[^ ^69^ ^]^ was used to render volumetric images.

### Measurement of Vessel Volume Occupancy and Vessel Density

To get the binary mask of the liver, adaptive thresholding was applied based on the three‐sigma (3*σ*) rule.^[^ ^70^ ^]^ Specifically, the post‐processed volumetric image block was first segmented at the peripheral of the field of view as the background signal and its mean absolute value *µ*_bg_ as well as the standard deviation *σ*_bg_ was calculated. Then *µ*_bg_ + 3*σ*_bg_ was used as the threshold to segment the binary liver mask, which was the region of interest (ROI) in this study. The volume of this binary mask was calculated as *V*_liver_. To segment the vessel binary mask, the 3*σ* thresholding of the Hessian‐filtered images were repeated within the ROI to get the binary mask. Its volume, denoted as *V*_vessel_, was then divided by the liver mask volume *V*_liver_ to get the VVO. To extract the vessel skeleton map, blood vessel skeletons were first extracted by generating vessel centerlines from the vessel segmentation masks using MATLAB's built‐in function bwskel. The vessel centerlines were broken into independent vessels at junction points. Then morphological cleaning was applied using MATLAB's built‐in function bwmorph to remove small independent vessels to reduce noise. The number of individual vessels *N*_vessel_ was calculated with MATLAB built‐in function bwlabeln, and the VD was calculated as *N*_vessel_/*V*_liver_.

### Measurement of Vessel Distribution Diversity and Morphological Irregularity

To calculate the vessel distribution diversity (VDD), a 1.2 mm × 1.2 mm × 1.2 mm sliding window was scanned across every voxel in the post‐processed volumetric image. For each image subset *M* ^sub^, the information entropy *E* was calculated within the window:

(1) $$E\left(M^{\mathrm{sub}}\right)=-\sum_{i=1}^{n}P_{i}\log_{2}P_{i}$$

where *n* = 256 denotes the number of discrete bins in the window, *P_i_* denotes the probability for a pixel to have a value that falls in the *i*th bin. The acquired entropy value was then assigned to the center pixel of the window, forming the VDD map *M*_E_. To measure the morphological irregularity (MI), the volumetric was first sliced along the *z* direction and a 3 mm × 3 mm sliding window was used to scan the *x*–*y* slice. The image subset was rotated from 0° to 180°, with a step size of 10°. At each rotation angle *θ*, the singular value decomposition (SVD) of the rotated image was calculated, acquiring the normalized SVD dominancy term *Σ*_11_ as a function of *θ*. After rotating the subset over 180°, the MI within the window was calculated as:

(2) $$A_{z}\left(M^{\mathrm{sub}}\right)=\exp\left[-\left(\max_{\theta }\Sigma_{11\left(\theta \right)}-\min_{\theta }\Sigma_{11\left(\theta \right)}\right)\right]$$

The same procedure was repeated for all the slices to get the MI map *A*_z_ along the *z* direction. Taking the *x* and *y* directions into consideration, the other two maps *A*_x_ and *A*_y_ were calculated, and the average of the three maps gave the final MI map *A*.

### Speed of Sound Estimation

To estimate the speed of sound (SoS) in the liver, a bulk of liver tissue was selected starting from ≈3 mm below the liver surface. DualSoS‐UBP algorithm was used to reconstruct the region of interest (ROI) with different tissue SoSes varying from 1400 to 1700 m s^−1^ with a step size of 10 m s^−1^. The SoS of the water was fixed and determined by temperature measurement during the experiment. 3D fast Fourier transform (FFT) was then applied on the volumetric image to acquire the image in the spatial frequency domain. The mean value of the absolute amplitude was used to estimate the image sharpness. The SoS that maximized the sharpness was used as the estimation. It represented the averaged SoS over the skin, the abdomen, and part of the liver, and could also be used in the dual‐SoS UBP reconstruction algorithm as the SoS of the tissue zone.

### Time Gating Algorithms for Motion‐Free Images

To recover the signals acquired during motion, the region after the surface signal was first selected as background and the 3*σ* rule was applied to distinguish the signals from the background. The first time index above the threshold at each azimuthal angle was selected to form the first‐arrival time array. Then a median filter (MATLAB built‐in function “medfilt1”) and Gaussian‐weighted moving average (MATLAB built‐in function “smoothdata”) were applied over different angles to smooth the time array, followed by numerical difference calculation. Finally, in the differential time arrays, the peak‐finding algorithms (MATLAB built‐in function “findpeaks”) were adopted to localize the peaks and troughs (by inversing the time array), thus determining the motion‐affected frames for removal. The remaining signals formed a motionless partial‐scan detection. Since the respiration cycles of the rat abdomen were spatially repeatable, the above procedures were repeated with two sequential 10‐s scans. The round‐trip signals were then summed to form the full‐scan signals. At angle positions where both forward and backward signals remained, the signals were averaged as the round‐trip signal. The authors finally interpolated at the angle positions where both forward and backward signals were removed. The final full‐scan signals were then used to reconstruct the motion‐free image.

### Time Gating Algorithms for Dynamic Images and Hemodynamics Analysis

The averaged peak‐trough distance was first calculated as the half respiratory cycle. Based on the observation that the breathing period remained stable within the round‐trip scanning time, the respiratory cycle was divided into multiple phases, the number of which was determined by the repetition period of the laser (20 ms). The signals were then repeatedly extracted from the same phase for the round‐trip scanning to reconstruct the dynamic images. Similar to the motion‐free images, the full‐scan images were averaged at the overlapping angular locations and interpolated at the missing ones. To mitigate aliasing artifacts caused by spatial under‐sampling in regions outside of the FOV, spatial interpolation and low pass filtered the PA signals were further applied before reconstruction. To get the vessel masks for hemodynamics analysis, a larger region was first segmented to isolate the blood vessel from other major vessels, and the segmentation masks were used through all the frames. Next, the 3*σ* rule was applied for adaptive hard thresholding at each frame to get a tighter vessel mask. Specifically, the square region was first segmented at the peripheral of the cross‐sectional images as the background signal and its mean absolute value *µ*_bg_ as well as the standard deviation *σ*_bg_ were calculated. Then *µ*_bg_ + 3*σ*_bg_ was used as the threshold to segment the binary vessel cross‐section mask.

### Statistical Analysis

To examine the significance of the difference between the healthy and obese rat livers, a one‐tailed Welch's (unequal variances) *t*‐test was performed on the vessel volume occupancy and vessel density.^[^ ^71^ ^]^ For angiographic irregularity, the mean AI value within the binary liver mask was used for comparison. The null hypothesis *H*_0_ for each *t*‐test was that the lean rat liver had less or equal population means as the corresponding obese rat liver. The results for the one‐tailed tests were shown as *p*‐values. Accordingly, the *p*‐values were generally less than 0.05, indicating a rejection of *H*_0_ at the 5% significance level. All statistical analysis was done via MATLAB's built‐in function “ttest2.”

### Imaging Protocols

All the animal experiments followed protocols were approved by the Institutional Animal Care and Use Committee (IACUC) of California Institute of Technology (IA20‐1737).

## Conflict of Interest

The authors declare no conflict of interest.

## Supporting information

Supporting Information

Supporting Information

## Data Availability

The data that support the findings of this study are available from the corresponding author upon reasonable request.

## References

[1] N. Chalasani , Z. Younossi , J. E. Lavine , M. Charlton , K. Cusi , M. Rinella , S. A. Harrison , E. M. Brunt , A. J. Sanyal , Hepatology 2018, 67, 328. 28714183 10.1002/hep.29367

[2] C. Estes , H. Razavi , R. Loomba , Z. Younossi , A. J. Sanyal , Hepatology 2018, 67, 123. 28802062 10.1002/hep.29466 PMC5767767

[3] Z. M. Younossi , A. B. Koenig , D. Abdelatif , Y. Fazel , L. Henry , M. Wymer , Hepatology 2016, 64, 73. 26707365 10.1002/hep.28431

[4] T. Marjot , A. Moolla , J. F. Cobbold , L. Hodson , J. W. Tomlinson , Endocr. Rev. 2020, 41, 66. 10.1210/endrev/bnz009 31629366

[5] O. Kucera , World J. Gastroenterol. 2014, 20, 8364. 25024595 10.3748/wjg.v20.i26.8364 PMC4093690

[6] S. L. Friedman , B. A. Neuschwander‐Tetri , M. Rinella , A. J. Sanyal , Nat. Med. 2018, 24, 908. 29967350 10.1038/s41591-018-0104-9 PMC6553468

[7] C. Alonso , D. Fernández‐Ramos , M. Varela‐Rey , I. Martínez‐Arranz , N. Navasa , S. M. Van Liempd , J. L. Lavín Trueba , R. Mayo , C. P. Ilisso , V. G. de Juan , M. Iruarrizaga‐Lejarreta , L. delaCruz‐Villar , I. Mincholé , A. Robinson , J. Crespo , A. Martín‐Duce , M. Romero‐Gómez , H. Sann , J. Platon , J. van Eyk , P. Aspichueta , M. Noureddin , J. M. Falcón‐Pérez , J. Anguita , A. M. Aransay , M. L. Martínez‐Chantar , S. C. Lu , J. M. Mato , Gastroenterology 2017, 152, 1449. 28132890 10.1053/j.gastro.2017.01.015 PMC5406239

[8] V. Vilgrain , M. Ronot , M. Abdel‐Rehim , M. Zappa , G. d'Assignies , O. Bruno , M.‐P. Vullierme , Diagn. Intervention Imaging 2013, 94, 713. 10.1016/j.diii.2013.03.010 23751229

[9] V. W.‐S. Wong , W.‐K. Chan , S. Chitturi , Y. Chawla , Y. Y. Dan , A. Duseja , J. Fan , K.‐L. Goh , M. Hamaguchi , E. Hashimoto , S. U. Kim , L. A. Lesmana , Y.‐C. Lin , C.‐J. Liu , Y.‐H. Ni , J. Sollano , S. K.‐H. Wong , G. L.‐H. Wong , H. L.‐Y. Chan , G. Farrell , J. Gastroenterol. Hepatol. 2018, 33, 70. 28670712 10.1111/jgh.13857

[10] M. Virarkar , A. C. Morani , M. Taggart , P. Bhosale , Semin. Ultrasound CT MRI 2021, 42, 381. 10.1053/j.sult.2021.03.003 34130850

[11] C. Aubé , F. Oberti , N. Korali , M.‐A. Namour , D. Loisel , J.‐Y. Tanguy , E. Valsesia , C. Pilette , M. C. Rousselet , P. Bedossa , H. Rifflet , M. Y. Maïga , D. Penneau‐Fontbonne , C. Caron , P. Calès , J. Hepatol. 1999, 30, 472. 10190731 10.1016/s0168-8278(99)80107-x

[12] J. Foucher , E. Chanteloup , J. Vergniol , L. Castéra , B. L. Bail , X. Adhoute , J. Bertet , P. Couzigou , V. de Lédinghen , Gut 2006, 55, 403. 16020491 10.1136/gut.2005.069153 PMC1856085

[13] J. Bercoff , G. Montaldo , T. Loupas , D. Savery , F. Mézière , M. Fink , M. Tanter , IEEE Trans. Ultrason. Ferroelectr. Freq. Control 2011, 58, 134. 21244981 10.1109/TUFFC.2011.1780

[14] C. Demené , T. Deffieux , M. Pernot , B.‐F. Osmanski , V. Biran , J.‐L. Gennisson , L.‐A. Sieu , A. Bergel , S. Franqui , J.‐M. Correas , I. Cohen , O. Baud , M. Tanter , IEEE Trans. Med. Imaging 2015, 34, 2271. 25955583 10.1109/TMI.2015.2428634

[15] Y. Hao , Q. Wang , Y. Yang , Z. Liu , Q. He , L. Wei , J. Luo , in 2019 IEEE Int. Ultrasonics Symp. (IUS), IEEE, Glasgow, Scotland 2019, p. 2263.

[16] S. B. Reeder , I. Cruite , G. Hamilton , C. B. Sirlin , J. Magn. Reson. Imaging 2011, 34, 729. 22025886 10.1002/jmri.22580 PMC3177109

[17] S. K. Venkatesh , M. Yin , R. L. Ehman , J. Magn. Reson. Imaging 2013, 37, 544. 23423795 10.1002/jmri.23731 PMC3579218

[18] B. Leporq , S. A. Lambert , M. Ronot , V. Vilgrain , B. E. Van Beers , NMR Biomed. 2014, 27, 1211. 25125224 10.1002/nbm.3175

[19] S. Singh , S. K. Venkatesh , R. Loomba , Z. Wang , C. Sirlin , J. Chen , M. Yin , F. H. Miller , R. N. Low , T. Hassanein , E. M. Godfrey , P. Asbach , M. H. Murad , D. J. Lomas , J. A. Talwalkar , R. L. Ehman , Eur. Radiol. 2016, 26, 1431. 26314479 10.1007/s00330-015-3949-z PMC5051267

[20] R. Girometti , A. Furlan , M. Bazzocchi , F. Soldano , M. Isola , P. Toniutto , D. Bitetto , C. Zuiani , Radiol. Med. 2007, 112, 394. 17440695 10.1007/s11547-007-0149-1

[21] S. J. Hectors , P. Kennedy , K.‐H. Huang , D. Stocker , G. Carbonell , H. Greenspan , S. Friedman , B. Taouli , Eur. Radiol. 2020, 31, 3805. 33201285 10.1007/s00330-020-07475-4

[22] D. Feier , C. Balassy , N. Bastati , J. Stift , R. Badea , A. Ba‐Ssalamah , Radiology 2013, 269, 460. 23878281 10.1148/radiology.13122482

[23] L. V. Wang , Med Phys 2008, 35, 5758. 19175133 10.1118/1.3013698 PMC2647010

[24] L. V. Wang , S. Hu , Science 2012, 335, 1458. 22442475 10.1126/science.1216210 PMC3322413

[25] J. Yao , J. Xia , L. V. Wang , Ultrason. Imaging 2016, 38, 44. 25933617 10.1177/0161734615584312 PMC4628905

[26] Y. Zhou , J. Yao , L. V. Wang , J. Biomed. Opt. 2016, 21, 061007. 27086868 10.1117/1.JBO.21.6.061007 PMC4834026

[27] L. Lin , P. Hu , X. Tong , S. Na , R. Cao , X. Yuan , D. C. Garrett , J. Shi , K. Maslov , L. V. Wang , Nat. Commun. 2021, 12, 882. 33563996 10.1038/s41467-021-21232-1 PMC7873071

[28] L. Li , L. Zhu , C. Ma , L. Lin , J. Yao , L. Wang , K. Maslov , R. Zhang , W. Chen , J. Shi , L. V. Wang , Nat. Biomed. Eng. 2017, 1, 0071. 29333331 10.1038/s41551-017-0071 PMC5766044

[29] L. Lin , L. V. Wang , Nat. Rev. Clin. Oncol. 2022, 19, 365. 35322236 10.1038/s41571-022-00615-3

[30] X. L. Deán‐Ben , T. F. Fehm , S. J. Ford , S. Gottschalk , D. Razansky , Light: Sci. Appl. 2017, 6, e16247. 30167242 10.1038/lsa.2016.247 PMC6062167

[31] Y. Asao , K. Nagae , K. Miyasaka , H. Sekiguchi , S. Aiso , S. Watanabe , M. Sato , S. Kizaka‐Kondoh , Y. Nakajima , K. Kishi , T. Yagi , Ultrason. Imaging 2022, 44, 96. 35549598 10.1177/01617346221099201 PMC9207988

[32] P. J. van den Berg , R. Bansal , K. Daoudi , W. Steenbergen , J. Prakash , Biomed. Opt. Express 2016, 7, 5081. 28018726 10.1364/BOE.7.005081 PMC5175553

[33] Y. Wu , S. Huang , J. Wang , L. Sun , F. Zeng , S. Wu , Nat. Commun. 2018, 9, 3983. 30266905 10.1038/s41467-018-06499-1 PMC6162313

[34] J. Lavaud , M. Henry , P. Gayet , A. Fertin , J. Vollaire , Y. Usson , J.‐L. Coll , V. Josserand , Int. J. Biol. Sci. 2020, 16, 1616. 32226306 10.7150/ijbs.40896 PMC7097915

[35] J. Lv , Y. Xu , L. Xu , L. Nie , Radiology 2021, 300, 204134. 10.1148/radiol.2021204134 33904773

[36] F. Oana , H. Takeda , K. Hayakawa , A. Matsuzawa , S. Akahane , M. Isaji , M. Akahane , Metabolism 2005, 54, 995. 16092047 10.1016/j.metabol.2005.02.016

[37] A. F. Frangi , W. J. Niessen , K. L. Vincken , M. A. Viergever , in Medical Image Computing and Computer‐Assisted Intervention — MICCAI’98, (Eds.: W. M. Wells , A. Colchester , S. Delp ), Springer, Berlin, Heidelberg 1998, p. 130.

[38] E. Ryschich , World J. Gastroenterol. 2004, 10, 3171. 15457566 10.3748/wjg.v10.i21.3171 PMC4611264

[39] C. M. Sehgal , G. M. Brown , R. C. Bahn , J. F. Greenleaf , Ultrasound Med. Biol. 1986, 12, 865. 3810981 10.1016/0301-5629(86)90004-9

[40] C. F. Chen , D. E. Robinson , L. S. Wilson , K. A. Griffiths , A. Manoharan , B. D. Doust , Ultrason. Imaging 1987, 9, 221. 3330336 10.1177/016173468700900401

[41] P. Bedossa , D. Dargère , V. Paradis , Hepatology 2003, 38, 1449. 14647056 10.1016/j.hep.2003.09.022

[42] M. E. Anderson , G. E. Trahey , J. Acoust. Soc. Am. 1998, 104, 3099. 9821351 10.1121/1.423889

[43] M. Jakovljevic , S. Hsieh , R. Ali , G. Chau Loo Kung , D. Hyun , J. J. Dahl , J. Acoust. Soc. Am. 2018, 144, 254. 30075660 10.1121/1.5043402 PMC6045494

[44] J. B. Boyd , G. I. Taylor , R. Corlett , Plast. Reconstr. Surg. 1984, 73, 1. 6197716 10.1097/00006534-198401000-00001

[45] L. Shekerdemian , D. Bohn , Arch. Dis. Child. 1999, 80, 475. 10208959 10.1136/adc.80.5.475 PMC1717913

[46] C. Crone , Acta Physiol. Scand. 1963, 58, 292. 14078649 10.1111/j.1748-1716.1963.tb02652.x

[47] T. C. Lee , R. L. Kashyap , C. N. Chu , CVGIP: Graph. Models Image Process. 1994, 56, 462.

[48] Y. Gazit , D. A. Berk , M. Leunig , L. T. Baxter , R. K. Jain , Phys. Rev. Lett. 1995, 75, 2428. 10059301 10.1103/PhysRevLett.75.2428

[49] A. Hahn , J. Bode , T. Krüwel , G. Solecki , S. Heiland , M. Bendszus , B. Tews , F. Winkler , M. O. Breckwoldt , F. T. Kurz , Sci. Rep. 2019, 9, 11757. 31409816 10.1038/s41598-019-47567-w PMC6692362

[50] A. Hammoutene , P.‐E. Rautou , J. Hepatol. 2019, 70, 1278. 30797053 10.1016/j.jhep.2019.02.012

[51] L. Lin , X. Tong , P. Hu , M. Invernizzi , L. Lai , L. V. Wang , Adv. Sci. 2021, 8, 2003396. 10.1002/advs.202003396 PMC8025032 33854889

[52] E. E. Powell , V. W.‐S. Wong , M. Rinella , Lancet 2021, 397, 2212. 33894145 10.1016/S0140-6736(20)32511-3

[53] A. Berzigotti , A. D. Gottardi , R. Vukotic , S. Siramolpiwat , J. G. Abraldes , J. C. García‐Pagan , J. Bosch , PLoS One 2013, 8, e58742. 23520531 10.1371/journal.pone.0058742 PMC3592829

[54] G. Targher , C. D. Byrne , A. Lonardo , G. Zoppini , C. Barbui , J. Hepatol. 2016, 65, 589. 27212244 10.1016/j.jhep.2016.05.013

[55] D. Viglino , I. Jullian‐Desayes , M. Minoves , J. Aron‐Wisnewsky , V. Leroy , J.‐P. Zarski , R. Tamisier , M. Joyeux‐Faure , J.‐L. Pépin , Eur. Respir. J. 2017, 49, 1601923. 28596431 10.1183/13993003.01923-2016

[56] A.‐C. Piguet , D. Stroka , A. Zimmermann , J.‐F. Dufour , Clin. Sci. 2009, 118, 401. 10.1042/CS20090313 19832698

[57] A. Katsarou , I. I. Moustakas , I. Pyrina , P. Lembessis , M. Koutsilieris , A. Chatzigeorgiou , World J. Gastroenterol. 2020, 26, 1993. 32536770 10.3748/wjg.v26.i17.1993 PMC7267690

[58] E. I. Neuschler , R. Butler , C. A. Young , L. D. Barke , M. L. Bertrand , M. Böhm‐Vélez , S. Destounis , P. Donlan , S. R. Grobmyer , J. Katzen , K. A. Kist , P. T. Lavin , E. V. Makariou , T. M. Parris , K. J. Schilling , F. L. Tucker , B. E. Dogan , Radiology 2018, 287, 398. 29178816 10.1148/radiol.2017172228

[59] E. Merčep , J. L. Herraiz , X. L. Deán‐Ben , D. Razansky , Light: Sci Appl 2019, 8, 18. 30728957 10.1038/s41377-019-0130-5 PMC6351605

[60] G.‐S. Jeng , M.‐L. Li , M. Kim , S. J. Yoon , J. J. Pitre , D. S. Li , I. Pelivanov , M. O'Donnell , Nat. Commun. 2021, 12, 716. 33514737 10.1038/s41467-021-20947-5 PMC7846772

[61] M. S. Hassouna , A. A. Farag , IEEE Trans. Pattern Anal. Mach. Intell. 2007, 29, 1563. 17627044 10.1109/TPAMI.2007.1154

[62] M. Li , C. Liu , X. Gong , R. Zheng , Y. Bai , M. Xing , X. Du , X. Liu , J. Zeng , R. Lin , H. Zhou , S. Wang , G. Lu , W. Zhu , C. Fang , L. Song , Biomed. Opt. Express 2018, 9, 1408. 29675292 10.1364/BOE.9.001408 PMC5905896

[63] Wikipedia , Near‐infrared window in biological tissue, https://en.wikipedia.org/wiki/Near-infrared_window_in_biological_tissue (accessed: 10, 2022).

[64] P. Hu , L. Li , L. Lin , L. V. Wang , IEEE Trans. Med. Imaging 2020, 39, 3535. 32746101 10.1109/TMI.2020.2998509 PMC7654731

[65] W. Kratzer , V. Fritz , R. A. Mason , M. M. Haenle , V. Kaechele , R. S. Group , J. Ultrasound Med. 2003, 22, 1155. 14620885 10.7863/jum.2003.22.11.1155

[66] R. Bulthuis , M. M. Dantuma , S. C. Kruitwagen , F. P. D. van Gameren , F. F. Lucka , B. B. D. Santi , S. S. Aarnink , L.‐F. de Geus‐Oei , B. T. Cox , S. S. Manohar , A. Javaherian , Eds., in Photons Plus Ultrasound: Imaging and Sensing 2022, vol. 11960, SPIE, Bellingham, WA 2022, p. PC119600X.

[67] American National Standards Institute, ANSI Z136.1‐2014 – American National Standard for Safe Use of Lasers, Laser Institute of America, Orlando, FL 2014.

[68] M. Xu , L. V. Wang , Phys. Rev. E 2005, 71, 016706. 10.1103/PhysRevE.71.016706 15697763

[69] S. Cho , J. Baik , R. Managuli , C. Kim , J. Photoacoust. 2020, 18, 100168. 10.1016/j.pacs.2020.100168 PMC7082691 32211292

[70] F. Pukelsheim , The American Statistician 1994, 48, 88.

[71] B. L. Welch , Biometrika 1947, 34, 28. 20287819 10.1093/biomet/34.1-2.28

