# Supplementary material for: Non‐Invasive 3D Photoacoustic Tomography of Angiographic Anatomy and Hemodynamics of Fatty Livers in Rats
Source: Adv Sci (Weinh). 2022 Nov 17;10(2):2205759. doi: 10.1002/advs.202205759 (PMC9839842; doi:10.1002/advs.202205759)
Supplement: Supplementary file 1 — Supporting Information [file ADVS-10-2205759-s002.pdf]

ADVANCED SCIENCE
Open Access

Supporting Information

for *Adv. Sci.*, DOI 10.1002/advs.202205759

Non-Invasive 3D Photoacoustic Tomography of Angiographic Anatomy and Hemodynamics of Fatty Livers in Rats

*Xin Tong, Li Lin, Peng Hu, Rui Cao, Yang Zhang, Joshua Olick-Gibson and Lihong V. Wang**

Supporting Information

Non-Invasive Three-Dimensional Photoacoustic Tomography of Angiographic Anatomy and Hemodynamics of Fatty Livers in Rats

*Xin Tong, Li Lin, Peng Hu, Rui Cao, and Lihong V. Wang**

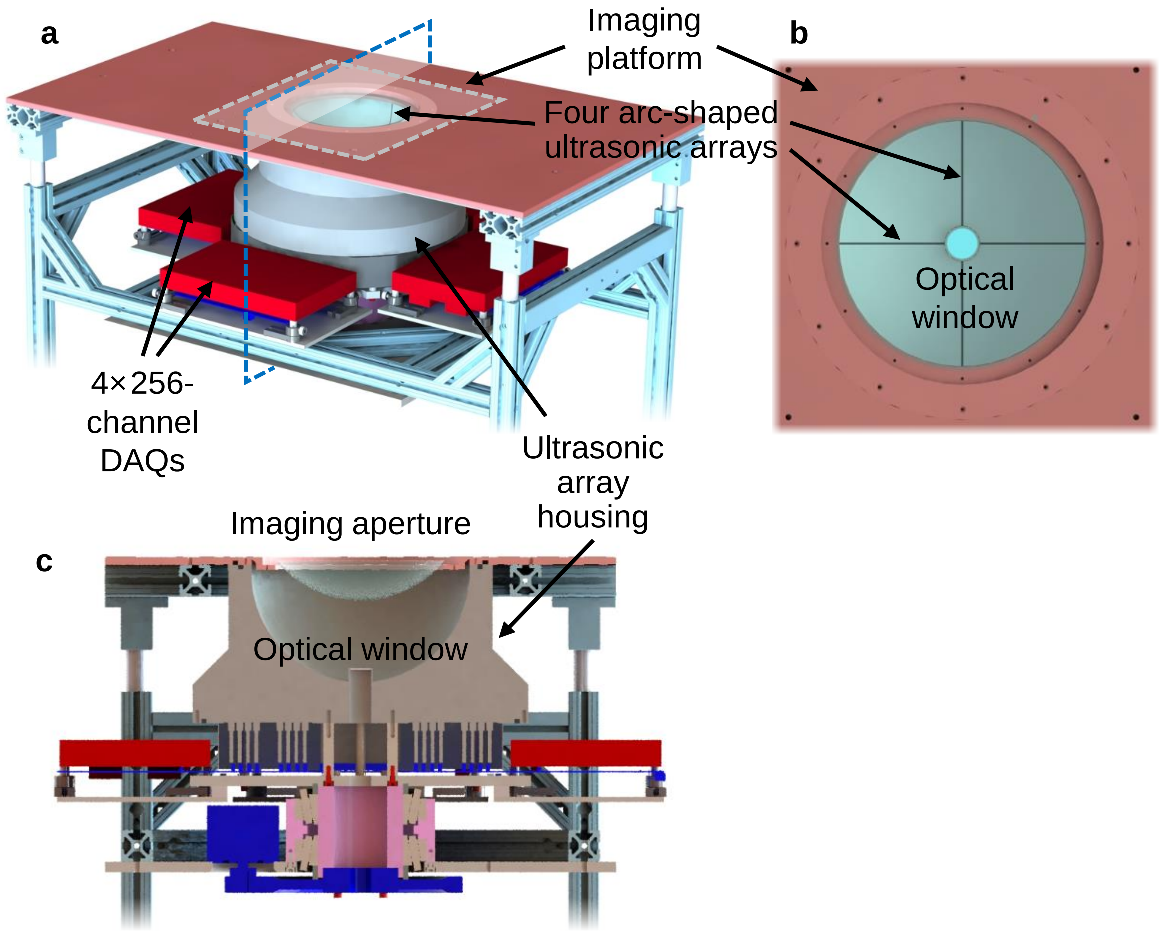

Supplementary Figure 1. Detailed schematics of the 3D-PAT system. a) Orthogonal projection of the system, showing the ultrasonic detection module, the hemispherical array housing the 4×256-channel DAQ circuits. For ultrasonic detection, four arc-shaped ultrasonic transducer arrays with a separation of 90 degrees were integrated into a hemispherical housing, as shown in b). c) Cross-sectional view of the 3D-PAT system, showing the hemispherical array housing as well as the optical window. The 1064 nm laser is shined towards the optical window before getting diffused for uniform illumination.

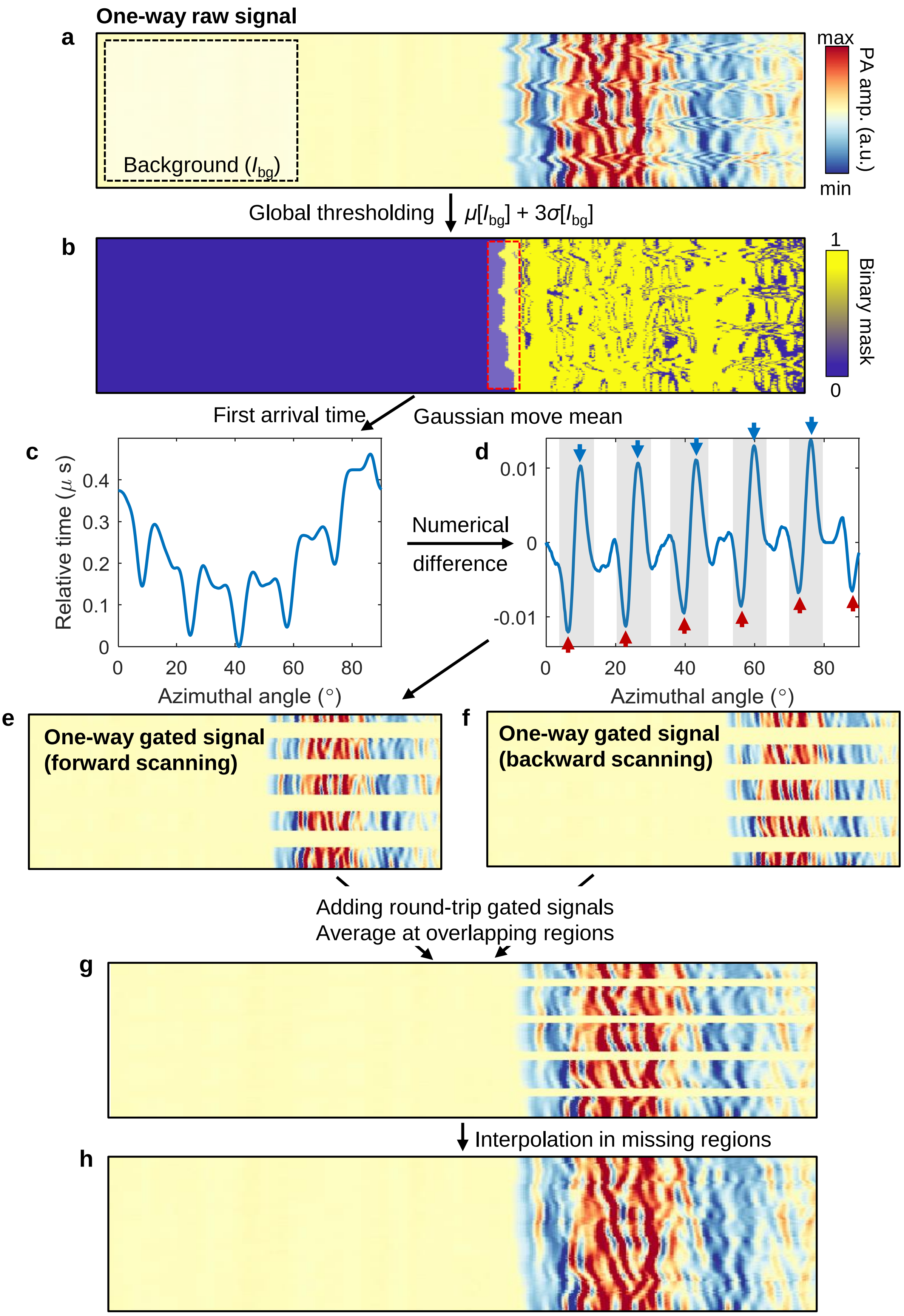
One-way raw signal
a
Background (I_{bg})
max
min
PA amp. (a.u.)
Global thresholding
$\mu[I_{bg}] + 3\sigma[I_{bg}]$
b
1
0
Binary mask
First arrival time
Gaussian move mean
c
Relative time (μ s)
0.4
0.3
0.2
0.1
0
0
20
40
60
80
Azimuthal angle (°)
Numerical
difference
d
0.01
0
-0.01
0
20
40
60
80
Azimuthal angle (°)
e
One-way gated signal
(forward scanning)
f
One-way gated signal
(backward scanning)
Adding round-trip gated signals
Average at overlapping regions
g
Interpolation in missing regions
h

Supplementary Figure 2. Schematics of the time gating pipeline. a) The region after the surface signal is selected as the background, from which the 3σ rule is applied to distinguish the signals from the background, forming the binary mask in b). c) The first time index above the threshold at each azimuthal angle to form the first-arrival time array after median filtration and Gaussian-weighted moving average. d) Numerical difference calculation presents the peaks and troughs and determines the motion-affected frames for removal. e) The remaining signals formed a motionless partial-scan detection. f) Similar procedure is applied for backward scanning signals. g) The round-trip signals are summed up to form the full-scan signals. At angle positions where both forward and backward signals remained, we averaged the signals as the round-trip signal. h) At the angle positions where both forward and backward signals are removed, spatial interpolation is applied to form the the final full-scan signals to reconstruct the motion-free image.

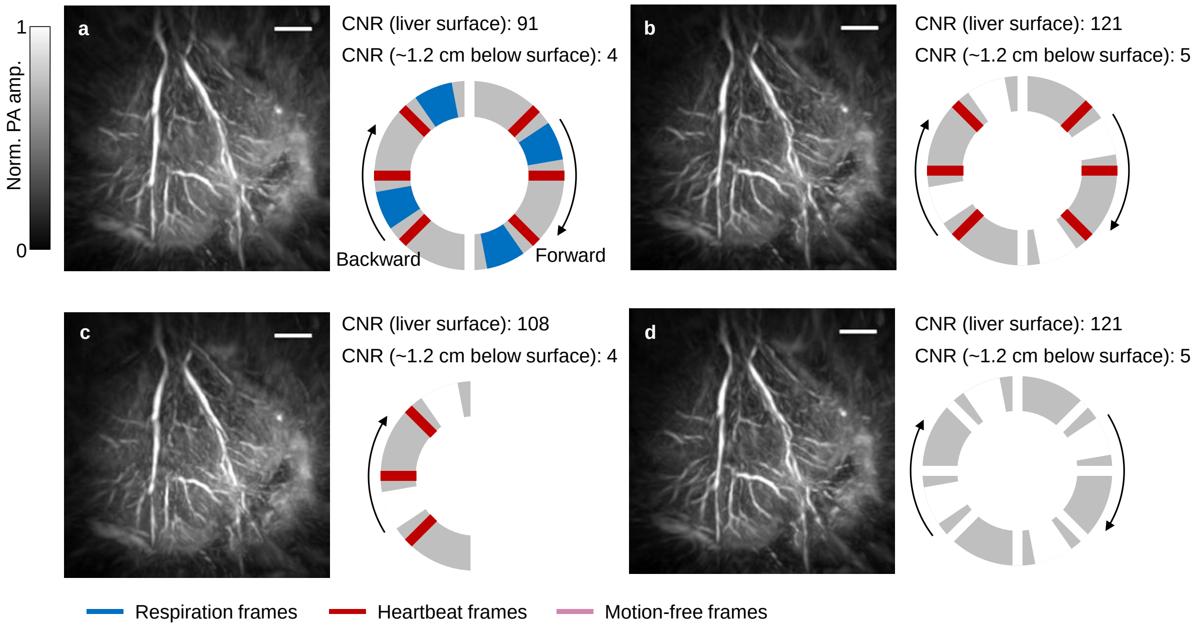

Supplementary Figure 3. Comparison between images with different time gating strategies. a) Coronal maximum amplitude projection (MAP) of a rat liver without time gating by round-trip scanning (20 seconds). The schematics of the scanning frames and CNRs at the liver surface and ~1.2 cm below the surface are noted. b) Coronal MAP of the rat liver with respiration gating by round-trip scanning. c) Coronal MAP of the rat liver with respiration gating by single-trip scanning. b) Coronal MAP of the rat liver with respiration and heartbeat gating by round-trip scanning. Scale bars, 5 mm.

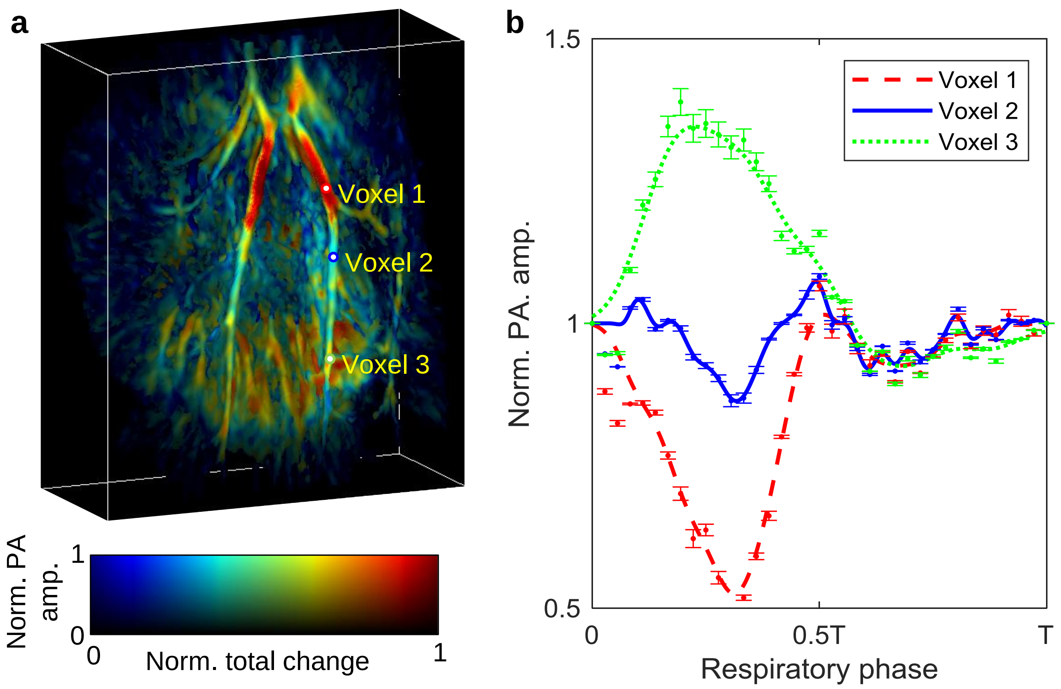

Supplementary Figure 4. Additional respiration-based time gating results. a) Total-signal-change-encoded orthogonal projection of a rat liver. b) Relative changes of the PA signals from three voxels in a). Data are normalized according to the first phase and plotted as means ± standard errors of the mean.

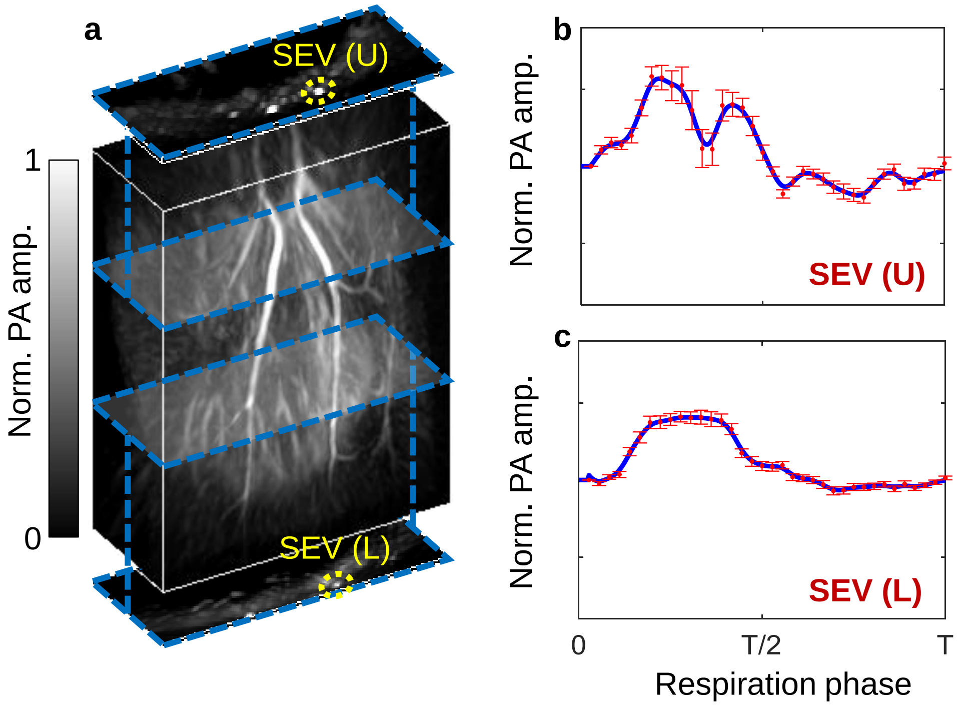

Supplementary Figure 5. Normalized total PA signal changes of a rat liver in a respiratory cycle. a) Orthogonal projection of a rat liver with two slices at different vertical positions showing the cross-sections of major blood vessels. SEV (U), upper (left) superior epigastric vessel; SEV (L), lower (left) superior epigastric vessel. b) – c) Total relative PA signal changes in the cross-sections during a respiratory cycle. The PA values are normalized with regard to the initial phases (denoted as 0 in *x* axis). Data are plotted as means ± standard errors of the mean.

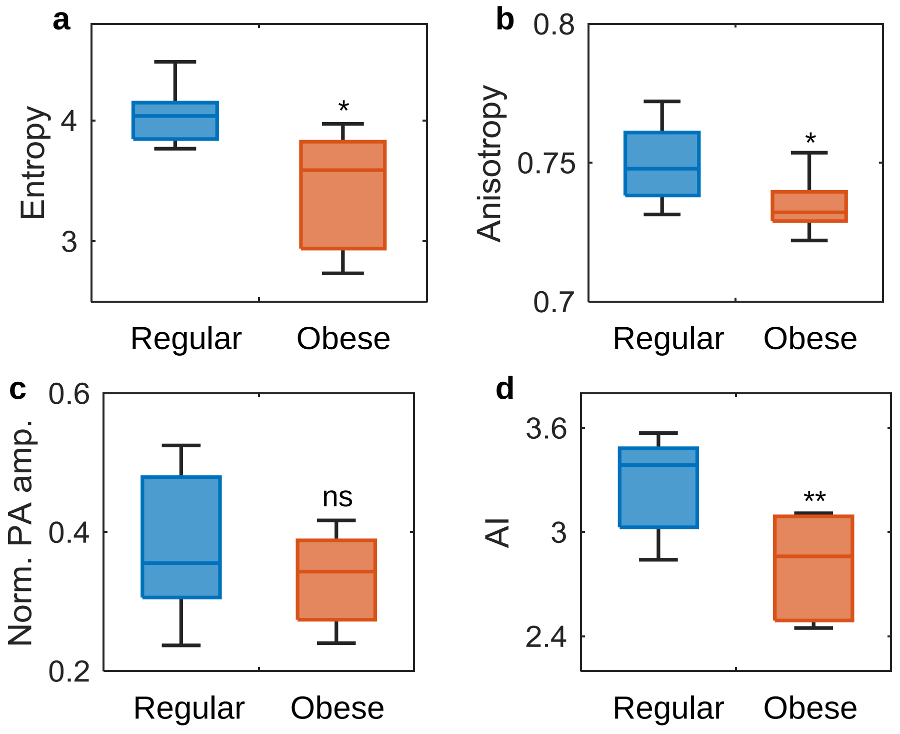

Supplementary Figure 6. Statistical comparison of entropy (a), anisotropy (b), normalized PA amplitude (c), and AI (with same binary masks, d) between lean and obese rat livers. Data are presented as boxplots; *p*-values are calculated using one-tailed Welch's (unequal variances) *t*-tests. * $p < 0.05$; ** $p < 0.01$; ns $p > 0.05$.
